# Active Game-Based Solutions for the Treatment of Childhood Obesity

**DOI:** 10.3390/s21041266

**Published:** 2021-02-10

**Authors:** Carina S. González-González, Nazaret Gómez del Río, Pedro A. Toledo-Delgado, Francisco José García-Peñalvo

**Affiliations:** 1 Grupo ITED, Universidad de La Laguna, 38200 San Cristóbal de La Laguna, Spain; petode@ull.edu.es; 2 Grupo GRIAL, Universidad de Salamanca, 37008 Salamanca, Spain; ngomrio@usal.es (N.G.d.R.); fgarcia@usal.es (F.J.G.-P.)

**Keywords:** active video games, exergames, childhood obesity, gamification

## Abstract

Obesity is one of the biggest health problems globally that, together with sedentarism, requires solutions that increase the enthusiasm towards physical activity. Therefore, this paper describes two solutions based on active games using the Kinect sensor and biometric sensors, designed for the outpatient treatment of childhood obesity. The solutions were applied in an intervention program based on active video games and motor games, developed with children in treatment for childhood obesity. An ad hoc questionnaire was used to assess the level of satisfaction, fun, learning, and behavior changes in the children of the experimental group that developed the intervention. The results showed a high index of satisfaction with the intervention program, as well as with the games developed. It is concluded that active video games and group games are highly motivating and can promote behavior change towards healthier life habits in children.

## 1. Introduction

Children and adolescents are currently surrounded by technology on the one hand, and with high rates of sedentarism on the other. In this sense, active video games can be beneficial to health, and promoting healthy living habits [1,2]. Active video games can be useful to increase children’s enthusiasm to physical activity [3]. Therefore, active video games are an excellent tool for improving children’s health and fighting obesity.

Some active video games allow for healthy exercise and fun at the same time. Some commercial video games, such as the Wii Fit, or Wii devices or Kinect, have been used in medicine [4]. Likewise, other health video games have been used to prevent, promote, and improve health, others for the improvement or training of health personnel [5,6].

Research has shown that children with obesity generally have low perceived sports self-efficacy and body dissatisfaction and tend to have a more sedentary life [7]. The first difficulty encountered by pediatricians in applying the prescription for a sport to children with obesity who are being treated is a lack of motivation [8]. Therefore, intervention programs are required to maintain children’s enthusiasm in the practice of physical activity [3,9].

On the other hand, there has been a popularization of biometric sensors in the sports sector. These devices, characterized by their reduced size, allow users to record physiological variables and other types of data related to sports practice. Currently, biometric sensors are part of an expanding market, and technology brands such as Apple and Samsung, not traditionally linked to sports, have become involved. Modern smartphones with biometric sensors and hardware are for sale, which allow connectivity with devices of some of the existing technologies. Different applications enable us to visualize the information collected or make use of it for other purposes. The two most popular wireless communication protocols for biometric data are ANT+ and Bluetooth Smart, characterized by low energy consumption and transmission in the ISM bands, open for industrial, scientific, and medical applications. These communication protocols allow data transmission from one or several biometric sensors to a smartwatch, smartphone, or any other device capable of storing and/or displaying such information. The diversity of measurable variables together with relatively low costs have made biometric sensors key elements in two main fields: on the one hand, they allow both professional and amateur athletes to keep track of the variables that affect performance during sports practice, optimizing training, and help to track progress; on the other hand, they allow people with health problems to keep track of their constants to keep track of the evolution of illness, predict dangerous situations, and keep health personnel informed of the patient’s condition. The fight against obesity implies, in addition to healthy nutrition, the incorporation of physical activity into the lifestyle. As an alternative to traditional exercise methods, there has been an increase in the market for so-called fitness games, more commonly known as exergames. Exergames are a type of video game oriented to promote the user’s physical activity, and the term derives from the combination of physical exercise and video games [10]. This goal is usually achieved by implementing direct interaction technologies in the interface that require the movement of large body extensions, making the player’s success depend on their degree of physical involvement. It is also increasingly common to incorporate these sensors to the sports environment (known as wearable sensors) and the development of biomechanical senses, which enable the analysis of the users [11,12]. 

Considering all the above, this paper presents two solutions based on active games created in the framework of a project called PROVITAO for the outpatient treatment of childhood obesity [9]. This project’s objective was to support the treatment of obesity at an early age through a model of game-based educational intervention designed for education in healthy habits. This paper seeks to answer the following research questions:

*RQ1. What is the level of satisfaction of children on active game-based solutions for the treatment of obesity?*

*RQ2. How does the educational intervention program based on active games influence children’s healthy habits?*

This paper is organized as follows. The related works are reviewed in Section 2. In Section 3, we present two active game-based solutions based on the Kinect and biometric sensors. Then, the methods and some results regarding the program of intervention are presented in Section 4 and Section 5, respectively. Finally, we summarize the conclusions and discussion in Section 6.

## 2. Related Works

There are several studies on video games and physical activity related to energy expenditure in educational programs carried out at school [12,13], which conclude that they increase children’s motivation to continue exercising outside of school [14]. However, the reduction in body weight depends on the frequency, duration, and intensity of the physical activity performed [15], and therefore not all active games are effective. Some studies have found that dance games’ cardiorespiratory response could be compared to medium to high-intensity aerobic dance [15]. On the other hand, active video games can promote healthy and active lifestyles, considerably increasing energy expenditure [16,17,18], comparable to other physical activities such as walking, jogging, or swimming [19,20]. Some commercial solutions, such as the active commercial platform (Nintendo Wii), have also proven to be effective for a more active life [9]. Other studies have demonstrated the effectiveness of exergames in promoting physical activity and calorie burning in children [21,22].

Despite the positive findings of several studies on energy expenditure with exergames, sports cannot be replaced because only a few active video games actually provide moderate intensity [21]. Additionally, not all exergames can maintain interest in the long term, for example, if performed as a routine or individual physical activity [19]. Interest and participation can be increased if games are played in group sessions [23].

Concern about sedentary lifestyles and their close relationship to the high rates of childhood obesity today acts as a catalyst for the development of new exergames and other forms of human–machine interaction and video game control variables, including heart rate [24]. As we have seen before, for a game of this type to be effective from a physiological point of view, it must demand a level of physical effort from the player that produces significant energy expenditure. In turn, overexertion, especially during prolonged periods, must be actively avoided from the game’s development to prevent an overdemand that affects the player’s satisfaction. However, there are physiological consequences of efforts close to or above the anaerobic threshold. According to [25], the use of non-conventional bio-controlled human–machine interfaces can result in performance or ergonomic benefits, among others. On the other hand, heart rate is valid when estimating physiological load during physical exercise and, in fact, according to Homan et al. [26], different studies show the existence of a linear relationship between the pulse and the intensity of submaximal exercise (i.e., below 85% of maximum heart rate). All of the above leads to the idea that using a heart rate monitor can help us improve the physiological effects of the game.

The idea of developing a game controlled almost entirely by the heart rate has been successfully carried out in various investigations. There are even applications on the market based on this principle [25,27,28]. The ones mentioned below are some examples:

Skip a Beat [27]. Skip a Beat is an application for smartphones with a heart rate monitor to train the user to control his heart rate. To do this, the game is divided into different levels according to the range of pulses. The pulses control the character’s size and movement (the Skip frog), and by keeping within the selected range, a higher final score is achieved. 

Press Masters Biathlon [25]. This prototype game, developed by researchers from the Helsinki University of Technology, consists of completing a ski slope in the shortest possible time, alternating with shooting tests. The heart rate is used, on the one hand, to control the speed of the run, so the higher the rate, the higher the speed; on the other hand, a higher pulse will negatively affect the accuracy of the shots. The main advantage of this type of interaction is that it can be played by practicing many different kinds of activities, such as a static bicycle or a race on site. However, the game is not specifically oriented to keep the players’ pulses within a range.

Flitz! [28] is an exergame in which the user must avoid the items of penalization and press the corresponding buttons of the controller (a platform composed of two columns with buttons at different heights) to attain the reward items. The game will be faster or slower depending on the player’s pulse and is the concept closest to being implemented in this study.

Next, two solutions created in the PROVITAO project [9] will be described as exergames for treating childhood obesity. 

## 3. Active Game-Based Solutions Based on Kinect and Biometric Sensors

The two solutions created use the Kinect sensor, a device capable of recognizing the human body and its environment, allowing interaction with information systems without maintaining physical contact with traditional control systems. The common use of Kinect and its libraries enable tracking the user’s joints in space, which makes it possible to control the avatar in the game using two fundamental methods. The first one consists of translating the tracked joints’ positions to the positions of the avatar’s joints in the virtual space. The main advantage of this method is a more detailed reproduction of the various positions adopted by the users within the sensor’s limitations. However, certain combinations of joint positions and sequences of movements (such as jumps) are not reproduced reliably enough, leading to overlapping limbs of the avatar, in addition to it not being possible to apply gravity effectively. 

The second method, the most widespread, is an indirect control of the avatar through gesture detection. In this case, tracking is used and compares the relative position of the joints of interest. If the joints pass through these positions within a specific time interval, then the gesture is considered complete.

The TANGO:H (Tangible Goals for Health) [24] platform uses the Kinect sensor, applying the human skeletal joint detection method and the cloud-based exercise creation tool. Another solution is describe, created to capture data from wearable biometric sensors and its integration with an exergame, using Kinect and applying the gesture detection method.

### 3.1. TANGO:H Web Designer

**Figure 1:**
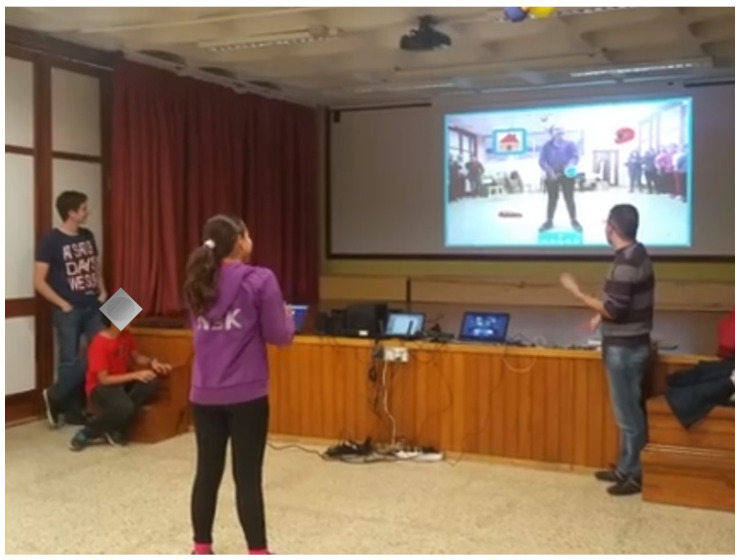
Execution of the exercises created in TANGO:H (Tangible Goals for Health) Designer.

The TANGO:H platform realizes exergames using the Kinect sensor. The TANGO:H platform has different player modes: the single-player mode, and the multiplayer mode. The sensor detects two human bodies simultaneously, and sequential, competitive, or collaborative exercises can be created in the multiplayer mode. The platform also has an integrated administrator that enables the management and grouping of users, and assigns exercises. Once the exercises are performed on the platform, they are stored to retrieve a statistical analysis of the results. The execution of the exercises can be assessed (Figure 1).

In addition to performing exercises, TANGO:H has an exercise designer, called TANGO:H Designer. This tool allows the design of exercises adapted to the users’ needs through a simple and intuitive interface. To make this design tool available without any installation and accessible from anywhere through the internet and a browser, a web solution has been developed for it. Hosted in a cloud server, this web solution allows the creation of and access to exercises. Additionally, the cloud platform must have user management, as well as its desktop version. The exercises created in both desktop and web versions are fully compatible and allow their execution in the TANGO:H client for final interaction with the user/patient.

The software allows the creation of different types of exercises: physical, cognitive, and free. In turn, cognitive exercises can be of matching, sorting, and classification genres. Each one of the exercise types has different interaction functionalities in the user interface. An exercise consists of a set of steps, and these, in turn, are organized into a set of phases. The phases are made up of a set of objectives with different joints of the human body’s skeleton associated with them, which will be the points of contact. For TANGO:H to interpret an exercise, the information must be structured as described above in an XML file. The hierarchy of the elements that make up the file structure is exercise, steps, phases, objectives, and the associated joints. Additionally, TANGO:H Designer uses multimedia files to design exercises, such as images or audios. 

This paper describes the application developed for the cloud, consisting of three fundamental modules: the user interfaces for interaction with the system; the database management module; and the server module that hosts the application. Each of these modules is implemented through different technologies. The graphic interface was implemented in HTML5 and CSS3, and the functionalities were implemented in JSP, JavaScript, and PHP. The database module was implemented in MySQL, and the server was implemented in XAMPP. 

**Figure 2:**
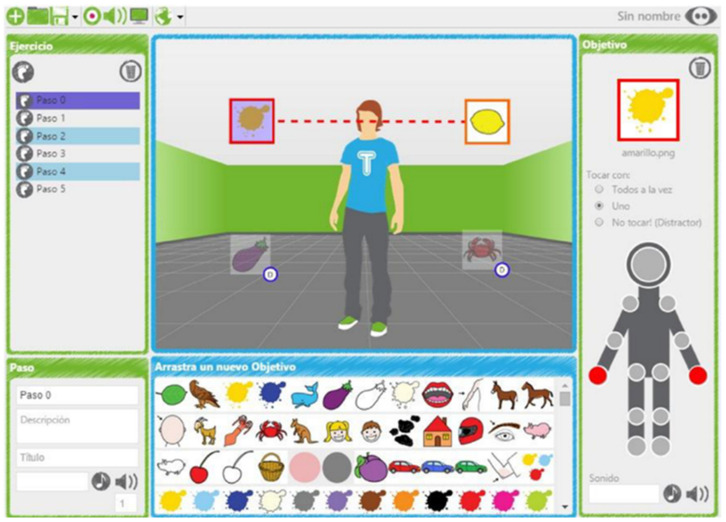
Exercise editing interface with its main elements.

The editing interface is the main screen of the application (Figure 2), because it is where the user defines the exercise’s parameters. This interface consists of the following elements:

(a) Menu bar: Executes the actions on the exercise file and the editor configuration.
(b) Exercise panel: Visualizes the outline of an exercise, allowing navigation through its components and performing actions on each of its elements.
(c) Target panel: View and edit the properties of the selected target.
(d) Available objectives panel: Contains objectives that can be used in creating an exercise.
(e) Design panel: Component where the exercise is designed, allowing the dragging of objectives to this panel.

The elements mentioned above are described in detail below.

*(a) Menu Bar*

The bar at the top of the window contains the access buttons to the application’s different options and tools. The elements available in this menu bar are explained in order from left to right.

- New. Create a new exercise. It directs the user to the selection window.
- Open. Opens an exercise from a file and loads it into the application. Only the exercises that belong to the user will be displayed.
- Save. It saves the current exercise.
- Objectives manager. Accesses the objective image management window.
- Sound manager. A sound management window is accessed.
- Background manager. The window for managing backgrounds is accessed.
- Languages. Selects the language preferences for the application.
- Information on the exercise. On the right side of the menu bar, information about the exercise being edited is displayed. Clicking on this information will open a window where the exercise’s name and description can be modified.

*(b) Exercise Panel*

This outlines the different components of an exercise: Steps, Phases, and Objectives. It allows the adding or removal of components and modifying some of their properties. This panel has the following components:

- Add new components. A set of controls located at the top of the panel. These enable the addition of a new step or phase. For cognitive exercises, the concept phase will not be used; each step will contain a set of objectives to be achieved.
- Step properties. In the lower part of the panel, there is a formula in which the selected Step properties are shown and edited. Here, the name, description, title, and audio file for the step can be edited.

*(c) Target Panel*

This panel displays and edits the properties of a Target. A Target presents color, sound, and behavior concerning the contact points: All at once, One, and Distractor. This panel has the following elements:

- Target and color. The upper part of the panel shows the target’s image, surrounded by a frame of the associated color.
- Contact points. The assignment of contact points to the lens is made on the character image shown at the bottom of the panel. The selected contact points are shaded with the color associated with the target. It is possible to update the behavior of the target using the controls located on the image.
- Sound of the target. At the bottom of the panel, there is a set of controls that facilitate sound to the target.
- Delete a lens. The control in the upper right corner of the panel allows the target to be removed from the exercise.

*(d) Available Objectives Panel*

Centered at the bottom of the main application window is the panel containing the set of images available as objectives (targets). These are the images available on the server. To add a new Objective to the exercise, the desired image is dragged to the Design Panel.

*(e) Design Panel*

The Design Panel refers to the arrangement and characterization of the objectives for each of the steps that make up the exercise which will be finally presented to the user on-screen. The basic actions that can be carried out on the Design Panel are the following:

- Insert target. To add a lens to the exercise, the desired image is dragged from the panel of available lenses to the day panel.
- Select a target (or several). A target is selected by clicking on it with the mouse. To select several targets at once, the “Ctrl” key is held while each of the desired targets are selected.
- Move one target. To move a target, it must be double-clicked and moved with the cursor around the Design Panel.

By clicking the right mouse button, a menu will be displayed with the following options:

- New step. It will add a new step to the structure of the exercise.
- Delete step. It will remove the current step from the exercise.
- Phase properties. It will access the properties of the selected phase.
- Matching (only for cognitive matching exercises). Allows pairing two selected objectives.

Having explored one of the active game-based solutions created to treat childhood obesity, in the next section, we will present another solution designed to integrate the biometric data provided by wearable sensors into an exergame. 

### 3.2. Exergame with Sensory Integration of Biometric Signals

Next, we will describe the solution created for the design of exercises in the cloud (web), as well as the design of a library of sensory integration for the creation of interfaces and registers of biometric signals.

The main sensory integration objective is to have an online measure of physical effort. Firstly, it can be used to modulate the activity to keep the user in the desired range of intensity. Secondly, the energy burn can be estimated for the user’s self-awareness. 

As mentioned above, the heart rate is a good indicator of the physical effort to which the body is subjected. The heart rate (HR) from which severe health damage can occur is known as the maximum heart rate (HRmax) and serves to calculate the intensity ranges at which we are working. Although we should subject everyone to a stress test for an accurate calculation of the HRmax, several formulas allow us to estimate it with some precision. Despite the accuracy of formulas of estimation when applied to children it is lower than with adults: compared to the traditional equation HRmax = 220−age, the equation HRmax = 208−(0.7 * age) has proven to be more accurate [29,30,31], and is recommended to be used in these circumstances [31]. Therefore, the latter was used in this project.

Depending on the maximum heart rate percentage, we can establish the intensity ranges shown in Table 1.

The cited study, carried out by Keytel et al., estimated an equation of prediction of energy expenditure (EE) from the pulse, age, sex, and body mass, and whose expression is:

EE = sex * (55.0969 + 0.6309 * HR + 0.1988 * weight + 0.2017 * age) + (1 − sex) * (−20.402 + 0.4472 * HR−0.1263 * weight + 0.074 * age) kJ/min

where sex = 1 for males and sex = 0 for females, HR is expressed in ppm, weight in kg, and age in years.

As with the maximum heart rate, an accurate calculation of energy consumption during physical activity can only be achieved by performing calorimetry tests on each individual. However, we can approximate indirect calorimetry using the heart rate because there is a linear relationship between the pulse and the energy expenditure in the range of approximately 90–150 ppm [33].

For the game to be executed correctly, it is a requirement to define a user profile in which the sex, weight, and age are specified. Secondly, it must be possible to connect with a heart rate sensor (pulsometer) through a USB–ANT+ interface.

The game’s main objective is to keep the user performing a physical effort (aerobic activity) within a range of healthy pulses. This is within the moderate zone defined by a HR in the range 60–70% of the HRmax. For this to happen, the user must perform specific exercises that involve the movement of large muscle groups, mainly the lower body, defined below:

- Jump. The central hip joint is raised above 12 cm from its previous position within 1.5 s.
- Squat. The center hip joint is lowered by six inches from its previous position in a 1.5 s interval.
- Lateral jumps. Starting from a position with the feet together (distance between the joints of the feet less than 30 cm) and finishing in this same position but having moved 25 cm to the right or left, depending on the response.
- Punch. The left or right hands advance in an interval of 1.5 s.

The game interface shows a small area of the scene in which the avatar is centered, and there are no more movements of the avatar except the lateral steps that must be executed to avoid certain objects. Overlaying this is a heads-up display (HUD) through which the player is provided with feedback on specific aspects of the game, such as:

- Heart Rate: Located in the upper right corner, it indicates the user’s heart rate, which changes color from blue to red depending on the range of heartbeats.
- Calories: Indicates the accumulated energy consumption since the beginning of the game and are based in the energy expenditure estimation explained above.
- Score: Fraction that indicates the number of objects drawn or destroyed satisfactorily concerning the total number of objects that have appeared.
- Time Left: Time to analyze the game.

To encourage the user to perform the exercises, the avatar is presented with a series of items that must be avoided or destroyed. When the user performs one of the actions, the avatar emulates it and, if it is one of the actions appropriate to the object, it will be successfully dodged or destroyed. Throughout the game, all the objects that have appeared as well as the number of them that have been drawn/deleted thanks to the actions executed by the user are counted; this relationship (presented as a fraction and percentage) is what we take here as a score. The objects and their corresponding action or actions are:

- Cylinders (“logs” or “barrels”): dodge by jumping.
- Blocks: destroy by frontal hit (punch).
- Shurikens: dodging by squats.
- Spheres: destroy by lateral jumps. They are presented in series and always appear in front of the user.

To keep the user intensity in the desired range, the following control loop has been implemented. As the user’s clicks move closer to the upper limit, the user will have to dodge fewer objects. On the other hand, when the user’s pulsations start to become too low, the number of objects to avoid increases. This situation is maintained until the game is analyzed once the programmed period has elapsed (five minutes). After this, the user has the option to retry or quit the game.

The mechanics of the game serve to keep the player active, and all its aspects are built directly or indirectly around this premise, advocating simplicity without forgetting that the goal is to demonstrate the usefulness of the incorporation of biometric sensors.

The start menu, besides preparing the user for the beginning of the game, has the function of guaranteeing that the intensity of the exercise is adequate and that the calories are estimated: on the one hand, knowing the age allows us to calculate the maximum heart rate, and with it the intensity intervals; on the other hand, in addition to age, we need to know the sex and weight of the user to estimate the energy expenditure. This is why there is also a specific range of permissible values for each field.

During the game, the avatar emulates the player’s movements as a mirror, which is considered the most natural method and allows better visualization of the objects because the avatar is facing the player.

Moreover, the HUD elements are arranged in such a way that the user can easily access the information, but with as little disruption to the game as possible.

Calculating the intensity intervals serves as a basis for controlling the rest of the game. Increasing or decreasing the frequency with which objects appear has no other purpose than intensifying or reducing the level of effort the user makes, respectively, to keep their heart rate within the ideal range.

Thus, the fact that the game score is represented as a proportion is a direct consequence of the fact that, on the one hand, the game takes place during a period previously assigned and, on the other hand, that the time between the appearance of two different obstacles depends on the player’s pulse because this means that during the programmed time, the number of total obstacles may vary.

At the same time, setting a period to play, without contemplating an end, responds to the indicated objective of keeping the player moving while maintaining simplicity.

**Figure 3:**
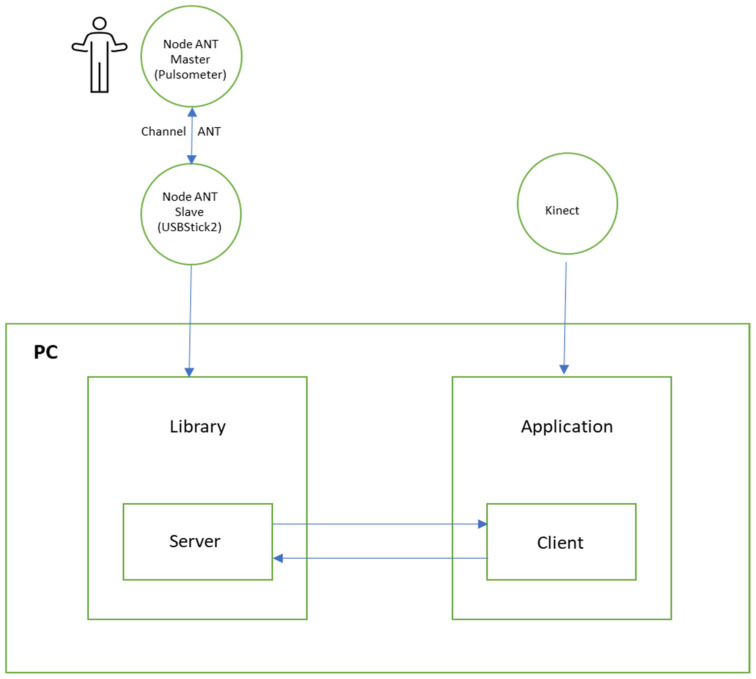
Simplified scheme of the adopted solution.

Different software has been used to implement the exergame, such as Visual C#, Visual C++, ANTware II 4.100 tools, SimulANT+ 1.6, Unity and the Kinect for Windows SDK. In addition, different hardware devices were used, such as the ANTUSB2 Sticks from Geonaute (two units), the Geonaute pulsometer, model SHRM1G, ANT+ technology and the Kinect sensor. Figure 3 presents a simplified diagram of the solution created.

**Figure 4:**
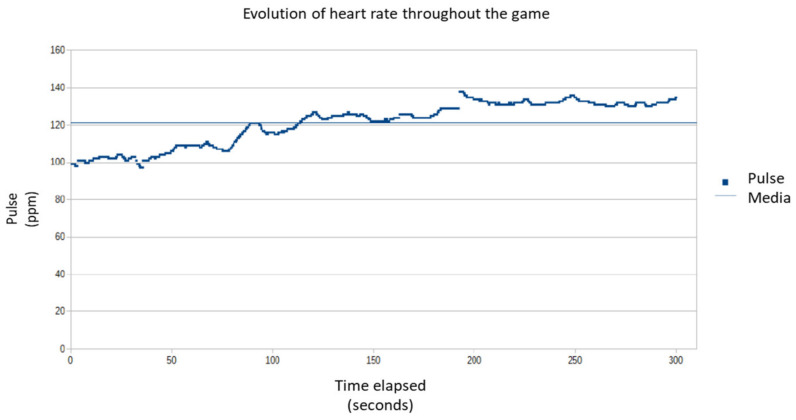
Evolution of the heart rate of a person playing the exergame.

**Figure 5:**
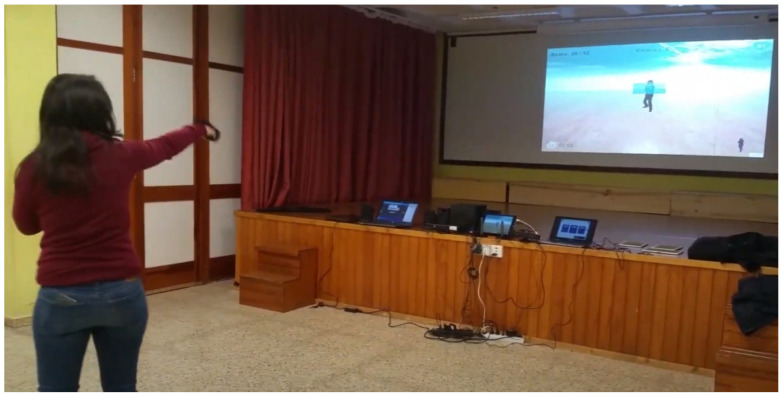
Subject playing exergame integrated with biometric sensors.

To test the effectiveness of the game, the heart rate of different subjects was recorded. The data analyzed corresponded to a five-minute game, requiring the participant to have not done any physical exercise in the previous hours. For example, Figure 4 shows the evolution of the heart rate of one of the subjects analyzed (Figure 5). In this case, the beginning of the aerobic zone is at 135 ppm. The initial heart rate is 99 ppm, and a slow but relatively constant increase is observed, reaching 120 ppm for the first time after 88 s. The pulses remain between 98 ppm at the beginning and a maximum of 138 ppm. The average is 121 ppm, somewhat lower than desired, although in this case, it neither exceeds the upper limit of the aerobic zone nor falls from the zone of moderate intensity.

The next section describes the method followed during the intervention program named PROVITAO.

## 4. Method

As mentioned above, the PROVITAO project aims to assist in the treatment of obesity in children by improving their life habits towards healthier behaviors [7]. The methodology followed in this study is quasi-experimental, with two annual phases. The sample is divided into an experimental group (children with obesity who participate in the intervention program) and a control group (children with obesity who do not participate in the intervention program). The target population was children diagnosed with obesity/type II diabetes seen in the hospital of reference. 

### 4.1. Participants

The sample consisted of 45 children between 6 and 12 years old (25 girls and 20 boys). The experimental group consisted of 25 children (15 girls and 10 boys), and the control group consisted of 20 children (10 girls and 10 boys). This convenience sample was selected using inclusion criteria to be diagnosed with childhood obesity (BMI > PC95; unit of measure kg/m^2^). The guidelines and ethical principles for medical research in humans, established in the Declaration of Helsinki, have been followed in implementing the project. Therefore, the knowledge and approval of the parents or guardians responsible for the children themselves were ensured. Furthermore, the research has been approved by the ethics committee of the University Hospital of the Canary Islands and by the ethics committee of the University of La Laguna.

### 4.2. Instruments

We have selected different instruments according to the areas of research of the project. All the questionnaires to be used in the project have been previously validated. Thus, the instruments and variables are described below:

- Biomedical area (pediatrics, nutrition, nursing)
  ➢ *Variables*: age, weight, height, BMI, skin folds, body perimeters, percentiles, blood pressure, and analytical parameters
  ➢ *Instruments:* weight, height meter, BMI formula, lipo calibre, inextensible tape measure, growth curve, sphygmomanometer, and blood analysis.
- Psychological and psycho-pedagogical area:
  ➢ *Variables* to be measured in children: evaluation of emotions resulting from human–computer interaction-observational measures; interpersonal relationships, relationships with parents, self-esteem, and self-confidence; knowledge and attitudes about healthy living habits and active video games.
  ➢ *Instruments*:
    - EMODIANA [34]. An instrument which allows measuring 10 basic emotions, represented with different expressions of a character associated with their corresponding labels, adjusted to children’s language. It is used during group intervention sessions.
    - BASC (Behavior Assessment System for Children and Adolescents) [35]. This is a multidimensional questionnaire which measures numerous aspects of behavior and personality.
    - Adaptation of the questionnaire on Physical-Sports Activity and Health-Wellness [36].
    - Player profile test. Adaptation of the questionnaire on use and attitudes towards video games [37].
    - Mediterranean Diet Quality Index—KIDMED Questionnaire (Mediterranean Diet Quality Index for children and teenagers) [38].
    - Questionnaires for children and parents ad hoc. Items to collect information about the intervention carried out during the project.

In this paper, we present some results of the questionnaire passed to children of the experimental group, due to this group having worked with active video games. 

### 4.3. Procedure

The intervention of the project was organized in different yearly phases and during the year in different moments. The first task was to select the sample and the diagnosis (medical and pre-tests) in both groups (experimental and control). Then, we carried out the yearly educational intervention according to the school’s academic course with the experimental group. The intervention was divided into three parts. The first consisted of different sessions developed for three months. During this part of the intervention, the researchers worked with children group sessions weekly. The learning goals of the intervention program were related to healthy habits. Additionally, regarding the game-based intervention, before starting the study, we collected and analyzed our sample’s game preferences using an adaptation of the Player profile test [37]. 

**Figure 6:**
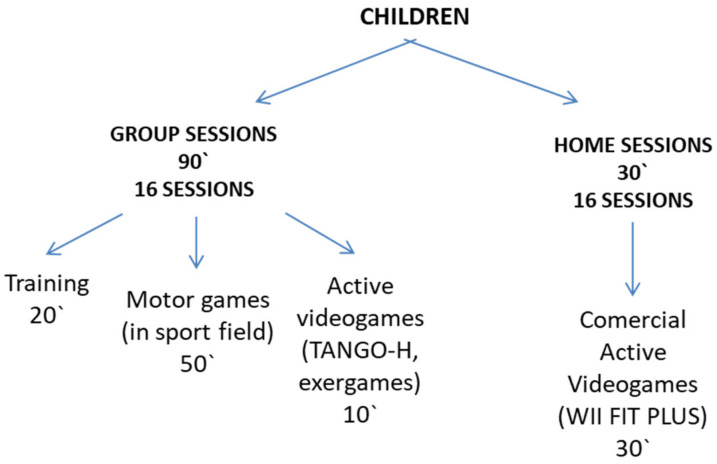
Structure of the intervention with the experimental group in PROVITAO.

Regarding the intervention carried out with the experimental group, the structure of sessions related to active video games can be seen in Figure 6.

The data collection was performed at the end of the intervention through a questionnaire created ad-hoc. The instrument consisted of 43 questions, mostly with a Likert scale from 1 to 5 on the level of agreement or disagreement with the questions, and open-ended questions to find out opinions about the intervention program’s aspects. The questionnaire’s main objective was to assess the level of satisfaction of the children with the educational intervention program in its different phases. Regarding active video games, variables such as satisfaction, fun—both in group sessions and at home—and comfort in the use of wearables were measured. We also sought to know how the intervention program influenced learning and behavior change in healthy living habits. The 43 items of the scale were independently measured from 1 (totally disagree) to 5 (totally agree) by each of the five experts. On the other hand, each item was scored in four predefined dimensions: (1) relevance of the question: the item is needed to the domain of study; (2) content adequacy: the extent to which the theme of the item reflects an important content of the field of study; (3) clear formulation: the use of a language that can be easily understood; and (4) target population addressing: how the items focus on the specific group of people (children) to which they are intended. The Kendall’s W non-parametric static was used to calculate the interrater agreement in the ordinal scale. Kendall’s W ranges from 0 (no agreement) to 1 (complete agreement). The results obtained showed a high agreement in the different dimensions: relevance of the question (0.88); content adequacy (0.76); clear formulation (0.73); and target population addressing (0.83). The researchers of the project collected the answers of children and explained the questions to them. The type of statistical methods used for data analysis for this instrument was descriptive statistics (frequency, mean and standard deviation). The main results of some variables analyzed through this instrument are presented below in Section 5.

Afterward, during the second part of the intervention, children designed a vocational project related to healthy habits. During this part of the intervention, the researchers developed group sessions with children and parents monthly. The last part of the intervention was developing the designed vocational project under the researchers’ supervision. During these moments, we collected data about different areas of the study using the instruments mentioned above. We have analyzed the results of questionnaires using SPSS 20.0. Afterwards, we conducted a comparative analysis using the obtained results of the different moments (pre/post) and groups.

Below are some results obtained in the educational intervention program using exergames, such as those described above, and motor games to support outpatient treatment of childhood obesity.

## 5. Results

In this section, we present some of the program’s main results concerning the active video games applied to the experimental group, diet, and the anthropometric variables measured during the intervention program related to obesity applied to both groups (experimental and control).

**Figure 7:**
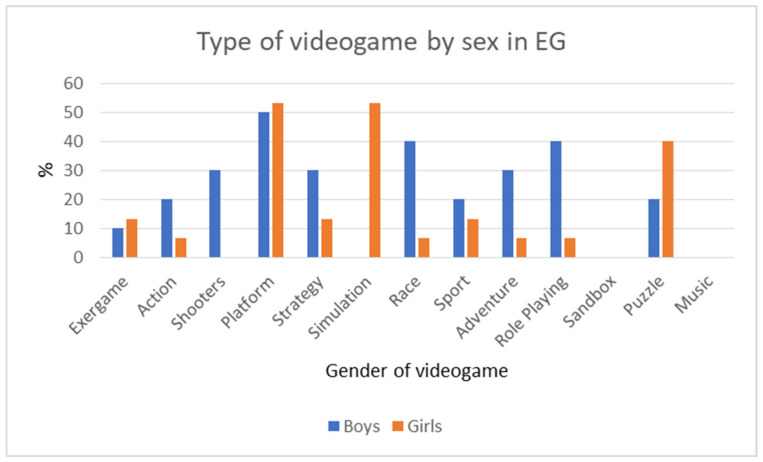
Preferences in the type of video games of the experimental group (EG) by sex.

As we can observe in Figure 7, there are some preferences about the type of video games between girls and boys. However, no significant differences have been found (boys 10.0% and girls 13.3%).

### 5.1. Perceived Variables Related to Active Video Games

This section presents the satisfaction questionnaire results about the PROVITAO intervention program [9] for the experimental group, which used active video games in the group sessions and individual homes, in addition to having developed weekly motor games.

*(a) Satisfaction*

**Figure 8:**
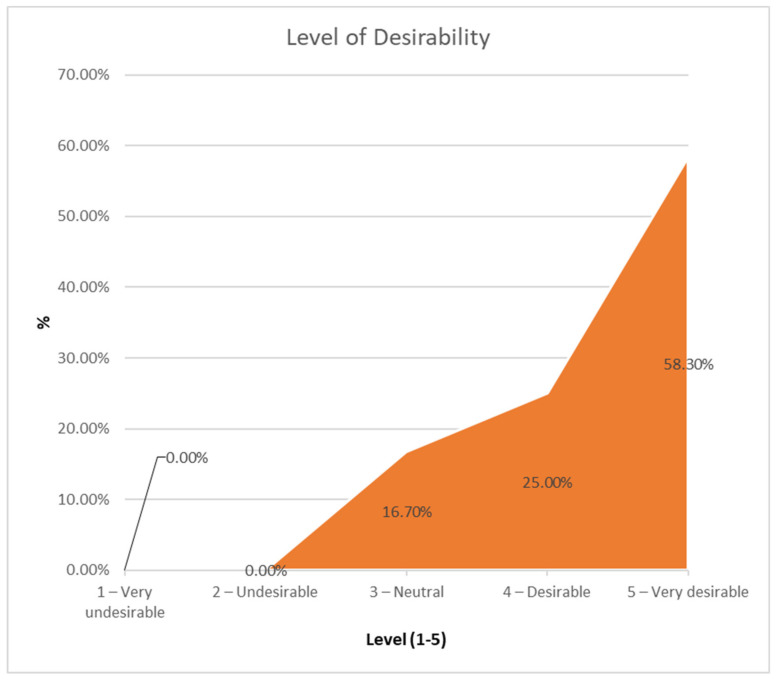
Answers to the question “Were you looking forward to the weekly sessions?”.

As shown in Figure 8, most of the children wanted to attend the weekly sessions of the intervention program (83.3%). On average, the level of desirability was 3.41 (1—very undesirable, 5—very desirable) (horizontal axis in Figure 8). No significant difference between girls (3.43) and boys (3.4) was found.

When asked what encouraged them to attend the weekly sessions, they replied as follows:

- What I learned (50%);
- Play (58.3%);
- Do physical activity (25%);
- Earn qualifying points (41.7%);
- Being with colleagues (50%);
- Being with the professionals (8.3%);
- Improve my lifestyle (25%).

Regarding the level of satisfaction with the training received in the group sessions, they said that they were moderately satisfied (25%), very satisfied (41.7%), and extremely satisfied (33.3%); there was a high level of satisfaction with the training received (75%). The level of satisfaction with the training on average was 4.08 (1—very unsatisfied, 5—extremely satisfied). No significant difference between girls (4.14) and boys (4.00) has been found.

On the other hand, they were asked about their level of satisfaction with the motor games held on the sports field, answering that they were moderately satisfied (8.3%), very satisfied (25%), and extremely satisfied (66.7%); 91.7% were highly satisfied with this part of the program. The level of satisfaction with the motor games on average was 4.58 (1—very unsatisfied, 5—extremely satisfied). No significant difference between girls (4.43) and boys (4.80) was found.

As for the active games, we asked participants what their level of satisfaction with TANGO:H was, with their answers being the following: moderately satisfied (16.7%), very satisfied (41.7%), and extremely satisfied (41.7%). Therefore, we observed a high satisfaction with TANGO:H (83.4%). The average level of satisfaction with TANGO:H was 4.25 (1—very unsatisfied, 5—extremely satisfied). No significant difference between girls (4.14) and boys (4.40) was found.

Concerning the sessions in the house carried out with the Wii, the level of satisfaction they showed was the following: moderately satisfied (33.3%), very satisfied (8.3%), and extremely satisfied (58.3%). The level of satisfaction with the Wii in the home was on average 3.25 (1—very unsatisfied, 5—extremely satisfied). No significant difference between girls (3.14) and boys (3.40) was found. We also asked them what motivated them to conduct the Wii sessions at home, and they answered the following:

- It was fun (58.3%);
- Do physical activity (25%);
- That it was mandatory (8.3%);
- Other (8.3%).

*(b) Fun*

**Figure 9:**
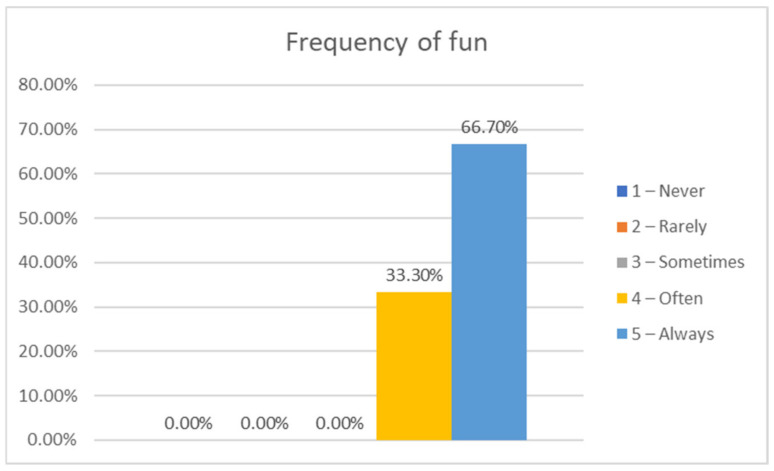
Answers to the question, “Did you have fun in the weekly sessions?”.

As for fun, we asked them if they had fun in the group sessions, answering often (33.3%) and always (66.7%); the enjoyment by the participants (Figure 9) was high (100%). The level of fun in the group sessions was on average 3.66 (1—never, 5—always). No significant difference between girls (3.71) and boys (3.60) was found.

Regarding what they liked best about the weekly group sessions, they answered the following reasons:

- The games and being with the companions;
- Because I had fun games;
- Improve my sessions and have a lot of fun;
- That I learned new things and had fun with all;
- To play;
- That I played and sweated and had a lot of fun;
- We learned;
- The games;
- Everything we did;
- The games were fun;
- That I had fun and had more friends to play;
- TANGO:H;
- The games we made.

**Figure 10:**
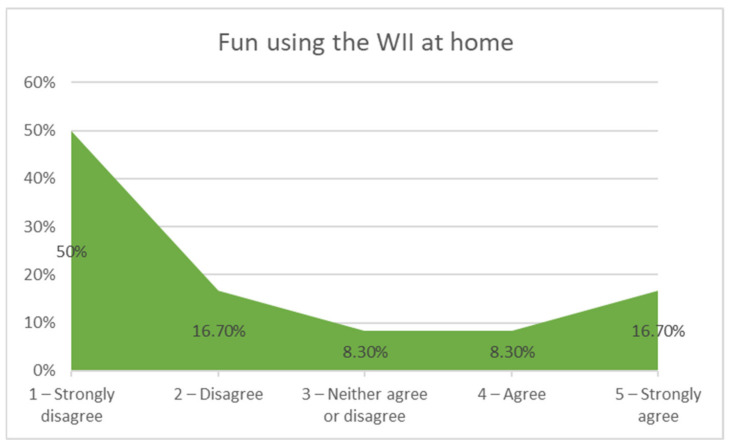
Answers to the question “Did you get bored of using the Wii at home?”.

As for the sessions at home, they were asked if they had become bored using the Wii. As shown in Figure 10, 66.7% disagreed with that statement, showing fun, but 25% did express boredom. Girls showed less boredom (90% disagreed with the question) using the Wii at home than boys (60% agreed to the question). They were asked, in the case that they had become bored, what had discouraged them, their answers being the following: always playing the same game (Fit plus) (50%), playing alone (37.5%) and other (12.5%).

*(c) Comfortability*

**Figure 11:**
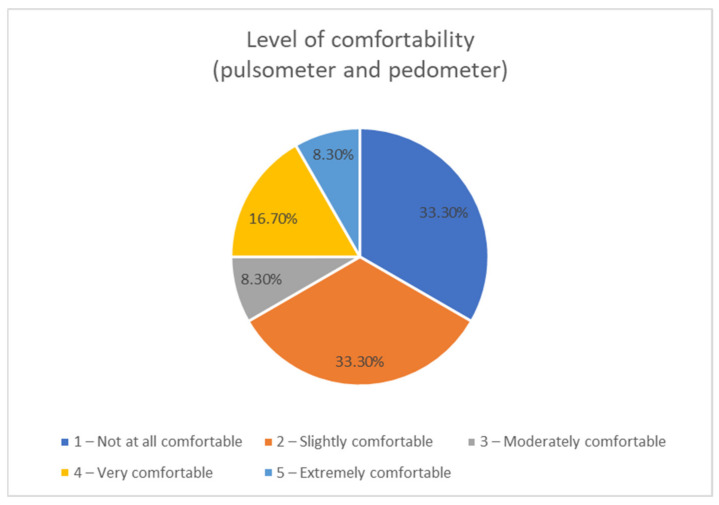
Answers to the question “Was it uncomfortable to wear the pulsometer (heart rate monitor) and pedometer?”.

Concerning the use of the wearables used in the intervention (wristwatch and Geonaute band), we asked them about the comfort level, and as can be seen in Figure 11, we saw that 66% stated that they had not been comfortable. The wearables’ level of comfort was on average 2.33 (1—not all comfortable, 5—extremely comfortable). Boys showed more tolerance to wearables (3.2) than girls (1.71).

*(d) Learning*

During the group sessions, we carried out training sessions about healthy habits (diet and physical activity), and all the games were designed around these topics. In terms of learning during the sessions, participants felt that they had learned moderately (16.7%), very (41.7%) and extremely well (41.7%). Therefore, we observed a high level of perceived learning (83.4%). Likewise, we asked them if they thought the learning obtained was useful, and 66.7% thought it was, 25% were neutral, and 8.3% disagreed with the statement. On the other hand, we asked them if they had learned about healthy living habits, with 91.7% answering that they had learned healthy living habits and 8.3% being neutral to this statement. The learning level on healthy habits was on average 3.58 (1—not at all, 5—extremely). No significant difference between girls (3.57) and boys (3.60) was found.

*(e) Behaviors*

**Figure 12:**
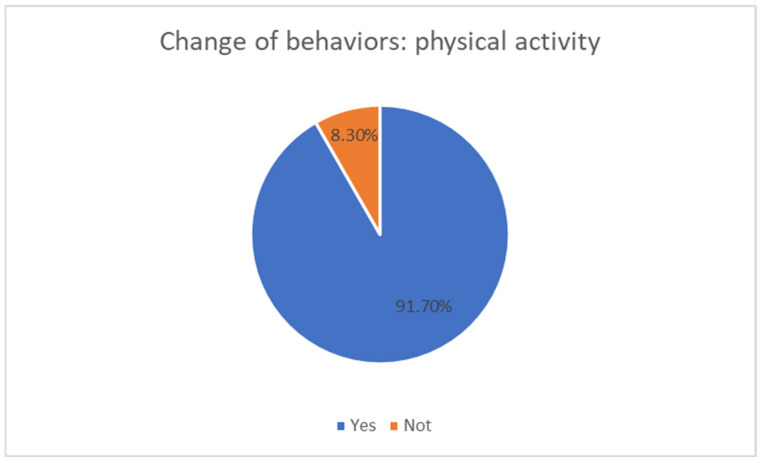
Answers to the question “Do you think the physical activity of the program has changed the way you behave for your physical activity habits?”.

In terms of behavior change concerning their physical activity habits, 91.7% responded that the intervention program had changed their habits (where 100% of boys and 85.7% of girls answered yes) (Figure 12). Likewise, 90.9% thought they had changed their diet behaviors (where 100% of boys and 85.7% of girls answered yes), now being more balanced and healthier. 

### 5.2. Emotions Related to the Intervention

The analysis of the emotions referred by children of the experimental group at the entry and exit of the group sessions gave us an indicator of the motivation with which they attended the intervention. However, we are especially interested in the informative nature of the emotional impact of the intervention.

To assess gamification’s emotional effect, the dynamics of play and training, we compared the emotions reported before starting the sessions with the emotions at the end. The emotions were registered with the Emodiana tool and have been categorized into positive, negative, and neutral [39]. 

All (100%) of the cases in which neutral emotions were reported at the session’s entry referred to positive emotions at the exit. Most (80%) of the entry’s negative emotions were modified to positive ones at the session’s exit, and 20% varied to neutral ones. A small number (4.3%) of the cases in which positive emotions reported at the entry changed to negative emotions, and the remaining cases maintained positive emotions. The statistical analysis confirmed that the differences in the distribution of the emotional categories were statistically significant both at the entry (χ^2^ (2) = 195.571; *p* ≤ 0.001) and at the exit (χ^2^ (2) = 206.333; *p* ≤ 0.001).

We also asked the participants to justify the referred emotions. The resulting categorization was subjected to the concordance analysis for more than two judges by means of Fleiss’ Kappa [40]. The resulting categorization, which achieved a Fleiss’ Kappa index of *k* = 0.84; an index considered excellent (greater than 0.75) according to Fleiss (1981). Most of the attributions included reasons related to the structure of the developed activity. Thus, we can see the positive impact of the group sessions in the experimental group’s children.

### 5.3. Anthropometric Variables Related to Obesity

No significant differences were found between the control and experimental groups or between the pre- and post-intervention on the anthropometric measures. We used, as a reference value of childhood obesity, the standardized growth charts of the Faustino Orbegozo Foundation, recommended by the Spanish Association of Paediatrics [41]. A BMI value of obesity at the age of 8.5 years is established as 23.5 for boys, and 24.7 for girls, and at the age of 9.5 years is 25.3 for boys and 25.6 for girls. Thus, we can see in Table 2 that both control and experimental groups reduced BMI after the intervention, but the weight decreased in the experimental group, while in the control group it increased. 

### 5.4. Healthy Habits

Regarding the impact of the program on healthy habits, particularly on their habits on feeding, we applied the KIDMED test as a tool to evaluate the adherence to the Mediterranean diet for children and youths [38]. The index ranges from 0 to 12, and is based on a 16-question test, administered by the researchers of the project. Questions denoting a negative connotation concerning the Mediterranean diet were assigned a value of −1, and those with a positive aspect +1. The sums of the administered test values were classified into three levels: (1) >8, optimal Mediterranean Diet; (2) 4–7, improvement needed to adjust intake to Mediterranean patterns; (3) ≤3, very low diet quality. The KIDMED index showed a slight improvement that was not significant in the experimental group, while the control group saw a worsening in its KIDMED index, which was significant (Table 3).

We started from a sample with homogeneous measures about the quality of the Mediterranean diet. Still, after the intervention, we found significant differences in the experimental group’s post-test and control (0.23). So, we can affirm that the means corresponding to the two groups are equal. This means that the children who participated in the experimental group managed to improve their diet quality [38].

## 6. Discussion

Regarding the research question RQ1 (What is the level of satisfaction of children on active game-based solutions for the treatment of obesity?), we can confirm that the designed investment program achieved a high satisfaction on the part of the children. A total of 83.7% wanted to attend the weekly sessions, mainly motivated by playing (58.3%), being with their peers (50%), learning (50%), and gaining points (41.7%). Therefore, we observe that the social factor of playing and gambling are fundamental elements for the satisfaction of minors. All (100%) of the children had fun in the group sessions, stating that they most enjoyed the games with their classmates and the active video games (TANGO:H). Likewise, satisfaction with other intervention program elements was evaluated, such as the training (75%) and the motor games (91.7%). TANGO:H was valued very positively (83.4%) in the group sessions. Additionally, the individual sessions in the homes with Wii were satisfactory (66.7%), although somewhat less than the group sessions, with the social aspect being what most motivated them to play the fun games (58.3%). However, they were also bored playing at home, mainly because they used the same game (Fit plus) (50%) and because they were alone (37.5%). 

We have analyzed the gender differences in the different variables. No significant difference between girls (3.43) and boys (3.4) has been found in the group sessions’ level of desirability. Additionally, no significant difference between girls (4.14) and boys (4.00) has been found in satisfaction with the training. Moreover, regarding the level of satisfaction with the motor games, no significant difference between girls (4.43) and boys (4.80) has been found. The level of satisfaction with TANGO:H was similar in girls (4.14) and boys (4.40), as was the use of Wii at home (girls (3.14) and boys (3.40). The group sessions’ level of fun was similar in girls (3.71) and boys (3.60). However, girls showed less boredom using the Wii at home than boys. Notably, in the pre-test, we found that only almost 10% of our sample played with the exergames, and children had other preferences in the types of video games. Boys prefer shooters, action, race, adventure, and role-playing video games, while girls like to play simulation games and puzzles. 

Additionally, we observed the emotions as an indicator of the motivation and interest with which children attended the weekly interventions. The emotions showed a positive impact on children and a high satisfaction to the group sessions. They have related positive emotions to the structure of the activity. Thus, we can see the positive impact of the group sessions on the experimental group’s children.

Concerning the research question RQ2 (How does the educational intervention program based on active games influence children’s healthy habits?) we can observe that the designed program was effective in the perceived learning of healthy living habits (91.7%), in addition to having changed their perceived behaviors for their physical activity habits (91.7%) and diet (90.9%).

Moreover, no significant difference between girls (3.57) and boys (3.60) has been found in the level of learning about healthy habits. However, through the KIDMED index we observed a slight improvement in the experimental group’s diet habits compared to the control group.

Besides, we measured anthropometric variables, such as BMI or weight, and no significant differences were found between the control and experimental groups or between the pre-and post-intervention stages. Both groups reduced BMI after the intervention, but the weight increased in the control group and decreased in the experimental group.

We also highlight that the use of biometric sensors with children is not always perceived as comfortable (66%), perhaps because of the bands’ use. We found that boys showed more tolerance to wearables (3.2) than girls (1.71). Besides, some children played with their wristwatches, and when a fault was detected, they would be taken out of the game to correct and reactivate the measurements. All this could influence their negative assessment of the use of these sensors.

## 7. Conclusions

This paper has presented different active game-based solutions based on the Kinect and biometric sensors for the treatment of childhood obesity in the framework of an educational intervention program called PROVITAO. Based on the obtained results, we conclude that active game-based interventions have a positive effect in the treatment of children with obesity. 

We can mention the following as the main contributions of this work:

- The creation of a web-based solution for creating active video games: TANGO:H designer, which allows for designing games in the cloud, and then be downloaded to be used with the Kinect sensor.
- The creation of a heart rate-controlled exergame that uses biometric sensors and the Kinect sensor.
- An educational intervention program approach to childhood obesity using commercially available active video games created by the research group, both at home and in weekly group sessions.

As the main limitations, we need to extend the program’s validation to more children and evaluate the biometric sensors’ effectiveness integrated into the exergames created. Moreover, validation is required from medical professionals as to the program’s efficacy in the long-term treatment of childhood obesity, especially when it comes to maintaining healthy behaviors. On the other hand, the coronavirus disease 2019 pandemic (COVID-19) [42,43,44] has affected the population’s physical activity and mental health [45]. Therefore, it is necessary to study whether this type of active video games can help maintain a healthy physical activity and at the same time, help people’s mental health. 

In conclusion, active video games and gamification can increase motivation toward physical activity, learning, and healthier living behaviors in children.

## Figures and Tables

**Table 1:** Intensity ranges according to the percentage of maximum heart rate (HRmax) [32].

Zone	Range	Characteristics
1	50–60% HRmax	Low intensity. Indicated for heating and recovery after exercise.
2	60–70% HRmax	Moderate intensity. Zone indicated for the increased metabolism of adipose tissue.
4	80–90% HRmax	Aerobic limit.
5	90–100% HRmax	Beginning of the anaerobic training zone (oxygen deficit).

**Table 2:** Anthropometric measures pre- and post-intervention of both groups.

Variables		Pre-Test		Post-Test	
		Median	SD	Median	SD
Age	Experimental Group	9.275	1.745	9.23	2.03
	Control Group	9.38	1.585	9.65	1.44
Weight	Experimental Group	60.615	13.95	58.915	11.435
	Control Group	55.865	10.69	56.335	7.575
Height	Experimental Group	1.425	0.115	1.435	0.115
	Control Group	1.445	0.11	1.465	0.09
BMI	Experimental Group	29.62	4.73	28.3	3.51
	Control Group	26.675	3.065	25.99	2.32
Waist-to-hip ratio	Experimental Group	0.98	0.055	0.995	0.07
	Control Group	0.91	0.15	1.55	2.28

**Table 3:** Index of KIDMED by groups and moments.

Group	Stage	KIDMED Index
Experimental Group Min = 5.00 Max = 11.00	Pre-test	7.67
Control Group Min = 4.00 Max = 11.00	Pre-test	7.11
Experimental Group Min = 5.00 Max = 10.00	Post-test	7.65
Control Group Min = 5.00 Max = 10.00	Post-test	6.78

## Data Availability

The data presented in this study are available on request from the corresponding author. The data are not publicly available due to privacy and ethical guidelines.

## References

[1] Baranowski T., Buday R., Thompson D.I., Baranowski J. (2008). Playing for real–video games and stories for health-related behavior change. Am. J. Prev. Med..

[2] Gonzalez C.S., Gómez N., Navarro V., Cairos M., Quirce C., Toledo P., Marrero-Gordillo N. (2016). Learning healthy lifestyles through active videogames, motor games and the gamification of educational activities. Comput. Hum. Behav..

[3] Guixeres J. (2011). Terapias en Obesidad Infantil: Estudio de los Videojuegos Activos Como Promotor del Ejercicio Físico. http://altorendimiento.com/terapias-en-obesidad-infantil-estudio-de-los-videojuegos-activos-comopromotor-del-ejercicio-fisico.

[4] Clark R.A., Bryant A.L., Pua Y., McCrory P., Bennell K., Hunt M. (2010). Validity and reliability of the Nintendo Wii Balance Board for assessment of standing balance. Gait Posture.

[5] Kato P.M. (2012). Evaluating Efficacy and Validating Games for Health. Games Heal. J..

[6] Sardi L., Idri A., Fernández-Alemán J.L. (2017). A systematic review of gamification in e-Health. J. Biomed. Inform..

[7] Southhall J.E., Okely A.D., Steele J. (2004). Actual and perceived physical competence in overweight and nonover-weight children. Pediatr. Exerc. Sci..

[8] Lu A.S., Kharrazi H., Gharghabi F., Thompson D. (2013). A systematic review of health videogames on childhood obe-sity prevention and intervention. Games Health Res. Dev. Clin. Appl..

[9] Del Rio N.G., González C.S.G., Gonzalez R.M., Adelantado V.N., Delgado P.T., Fleitas Y.B. Gamified educational programme for childhood obesity. Proceedings of the 2018 IEEE Global Engineering Education Conference (EDUCON).

[10] Cuberos R.C., Gracés T.E., Fernándes Á.C., Sánches M.C., López Fernándes J.F., Ortega F.Z. (2015). Exergames para la Mejora de la Salud en Niños y Niñas en Edad Escolar: Estudio a Partir de Hábi-tos Sedentarios e Índices de Obesidad. http://relatec.unex.es/article/view/1471.

[11] Gao Z., Chen S. (2014). Are field-based exergames useful in preventing childhood obesity? A systematic review. Obes. Rev..

[12] González C.S., Gómez del Río N.G., Navarro V. (2018). Exploring the benefits of using gamification and videogames for physical exercise: A review of state of art. IJIMAI.

[13] Papastergiou M. (2009). Exploring the potential of computer and video games for health and physical education: A literature review. Comput. Educ..

[14] Sánchez López A.M., Aguilar Cordero M., González Jiménez E., Padilla López C., Álvarez Ferre J., Ocete Hita E. (2011). La Obesidad como factor pronóstico de la falta de motivación en el niño y en el adolescente. X Congreso Nacional de la SEEDO. Revista Española de la Obesidad.

[15] Tan B., Aziz A., Chua K., The K. (2002). Aerobic demands of the dance simulation game. Int. J. Sports Med..

[16] Lanningham-Foster L., Jensen T., Foster R., Redmond A., Walker B., Heinz D. (2006). Energy expenditure of sedentary screen time compared with active screen time for children. J. Pediatr..

[17] Lanningham-Foster L., Foster R.C., McCrady S.K., Jensen T.B., Mitre N., Levine J.A. (2009). Activity-promoting video-games and increased energy expenditure. J. Pediatr..

[18] Mellecker R., McManus A. (2008). Energy expenditure and cardiovascular responses to seated and active gaming in chil-dren. Arch. Pediatr. Adolesc. Med..

[19] Maddison R., Mhurchu C., Jull A., Jiang Y., Prapavessis H., Rodgers A. (2007). Energy expended playing video console games: An opportunity to increase children’s physical activity?. Pediatr. Exerc. Sci..

[20] Wetzsleon R., Swanson K., Pickett K. (2008). Energy expenditure and ground reaction forces of an active video game, Dance Dance Revolution, in healthy weight and overweight children. Med. Sci. Sports Exerc..

[21] Staiano A.E., Beyl R.A., Guan W., Hendrick C.A., Hsia D.S., Newton R.L. (2018). Home-based exergaming among children with overweight and obesity: A randomized clinical trial. Pediatr. Obes..

[22] Brown T., Moore T.H., Hooper L., Gao Y., Zayegh A., Ijaz S., Elwenspoek M., Foxen S.C., Magee L., O’Malley C. (2019). Interventions for preventing obesity in children. Cochrane Database Syst. Rev..

[23] Chin A., Paw M., Jacobs W., Vaessen E., Titze S., van Mechelen W. (2008). The motivation of children to play an active video game. J. Sci. Med. Sport.

[24] Del Río N.G., González-González C.S., Toledo-Delgado P.A., Muñoz-Cruz V., García-Peñalvo F. (2020). Health Promotion for Childhood Obesity: An Approach Based on Self-Tracking of Data. Sensors.

[25] Nenonen V., Lindblad A., Häkkinen V., Laitinen T., Jouhtio M., Hämäläinen P. (2007). Using heart rate to control an interactive game. Proceedings of the SIGCHI Conference on Human Factors in Computing Systems.

[26] Hoffmann K., Wiemeyer J., Hardy S., Göbel S. (2014). Personalized Adaptive Control of Training Load in Exergames from a Sport-Scientific Perspective. Proceedings of the Constructive Side-Channel Analysis and Secure Design.

[27] Happitech Skip a Beat Game. http://skipabeatgame.com/.

[28] Osterwalder F., Pollinger P. Flitz! Adaptative exErgame. http://www.echo-grafik.ch/flitz/index.html.

[29] Machado F., Denadai B. (2011). Validity of maximum heart rate prediction equations for children and adolescents. Ar-Quivos Bras. Cardiol..

[30] Roy S.J. (2009). Validation of Maximal Heart Rate Regression Equations. Ph.D. Thesis.

[31] Cicone Z.S., Holmes C.J., Fedewa M.V., Macdonald H.V., Esco M.R. (2019). Age-Based Prediction of Maximal Heart Rate in Children and Adolescents: A Systematic Review and Meta-Analysis. Res. Q. Exerc. Sport.

[32] Benson R., Connolly D. (2019). Heart Rate Training.

[33] Keytel L.R., Goedecke J.H., Noakes T.D., Hiiloskorpi H., Laukkanen R., van der Merwe L., Lambert E.V. (2005). Prediction of energy expenditure from heart rate monitor-ing during submaximal exercise. J. Sports Sci..

[34] González-González C.S., Cairós-González M., Navarro-Adelantado V. (2013). EMODIANA: Un instrumento para la evaluación subjetiva de emociones en niños y niñas. Actas del XIV Congreso Internacional de Interacción Persona-Ordenador.

[35] Reynolds C.R., Kamphaus R.W. (2004). BASC-2. Behavior Assessment System for Children.

[36] Estévez-López F., Tercedor P. (2012). Delgado-Fernández, M. Recomendaciones de actividad física para adultos sanos. J. Sport Health Res..

[37] Alfageme González M.B., Sánchez Rodríguez P.A. (2003). Un instrumento para evaluar el uso y las actitudes hacia los videojuegos. Pixel-Bit. Rev. Med. Educ..

[38] Del Río N.G., González-González C.S., Martín-González R., Navarro-Adelantado V., Toledo-Delgado P., García-Peñalvo F. (2019). Effects of a Gamified Educational Program in the Nutrition of Children with Obesity. J. Med Syst..

[39] González-González C.S., Navarro-Adelantado V. Methods and techniques for evaluating the emotional expe-riences of children with active videogames. Proceedings of the XVI International Conference on Human Computer Interaction.

[40] Fleiss J.L., Levin B., Paik M.C. (1981). The measurement of interrater agreement. Stat. Methods Rates Pro-Portions.

[41] Fernández C., Lorenzo H., Vrotsou K., Aresti U., Rica E., Sánchez I. (2011). Estudio de crecimiento de Bilbao. Cur-vas y tablas de crecimiento. https://www.fundacionorbegozo.com/wp-content/uploads/pdf/estudios_2011.pdf.

[42] Fardoun H., González-González C.S., Collazos C.A., Yousef M. (2020). Estudio exploratorio en Iberoamérica sobre procesos de enseñanza-aprendizaje y propuesta de evaluación en tiempos de pandemia. Educ. Knowl. Soc..

[43] García-Peñalvo F.J., Corell A., Abella-García V., Grande-de-Prado M. (2020). Online Assessment in Higher Education in the Time of COVID-19. Educ. Knowl. Soc..

[44] García-Peñalvo F.J., Corell A. (2020). La COVID-19: ¿enzima de la transformación digital de la docencia o reflejo de una crisis metodológica y competencial en la educación superior?. Campus Virtuales.

[45] Violant-Holz V., Gallego-Jiménez M.G., González-González C.S., Muñoz-Violant S., Rodríguez M.J., San-sano-Nadal O., Guerra-Balic M. (2020). Psychological Health and Physical Activity Levels during the COVID-19 Pan-demic: A Systematic Review. Int. J. Environ. Res. Public Health.

